# Associations of Parental Education With Children’s Infectious Diseases and Their Mediating Factors: The Japan Environment and Children’s Study (JECS)

**DOI:** 10.2188/jea.JE20240192

**Published:** 2025-04-05

**Authors:** Masami Narita, Midori Yamamoto, Kenichi Sakurai, Chisato Mori, the Japan Environment and Children’s Study Group

**Affiliations:** 1 Department of Sustainable Health Science, Graduate School of Medical and Pharmaceutical Sciences, Chiba University, Chiba, Japan; 2 Department of Recruit and Career Development, Ono Pharmaceutical Co., Osaka, Japan; 3 Department of Sustainable Health Science, Center for Preventive Medical Sciences, Chiba University, Chiba, Japan; 4 Department of Nutrition and Metabolic Medicine, Center for Preventive Medical Sciences, Chiba University, Chiba, Japan; 5 Department of Bioenvironmental Medicine, Graduate School of Medicine, Chiba University, Chiba, Japan

**Keywords:** parental education, child’s infection, mediating factor, vaccination

## Abstract

**Background:**

Parents’ educational background is presumed to influence the incidence of vaccine-preventable diseases in children through their decisions about vaccinations and other family lifestyle choices. Regarding voluntary vaccination, a household’s economic situation may also be associated with non-vaccination. Therefore, this study investigated the association between parental education and vaccine-preventable diseases (varicella, mumps, influenza [flu], pertussis, measles, and rubella) in children, which currently remains elusive.

**Methods:**

We used datasets from the Japan Environment and Children’s Study, which included 104,062 fetal records; our study population comprised 80,930 children up to the age of 3 years. The associations between parental educational background and children’s infectious diseases were examined using binomial logistic regression analysis. The mediating effects of household income, vaccination, and smoking were examined using a path analysis.

**Results:**

For varicella, mumps, and influenza covered by voluntary vaccination, a higher education level of the father was associated with a lower incidence of infection. The association between mothers’ education and children’s infection was limited. There were both income-mediated and non-income-mediated pathways between parental education and voluntary vaccination. For pertussis, measles, and rubella, which are covered by routine vaccines, there was no association between parental education and the child’s infection.

**Conclusion:**

An association between parental education and childhood infections was observed. Providing financial support for vaccination and communicating the benefits of vaccination in a way that parents at all levels of education can understand may help reduce the incidence of infectious diseases among children.

## INTRODUCTION

To protect children’s health, a healthy lifestyle, including a well-balanced diet, adequate exercise, sufficient sleep, a safe environment, and preventive medical care, such as health checkups, is necessary. Children’s lifestyle, living environment, and preventive care greatly depend on parental awareness and behavior. Higher education has been reported to be positively associated with health behaviors,^1^^,^^2^ suggesting that parents’ educational backgrounds play a role in shaping their children’s health behaviors.

Common infectious diseases in children, including pertussis, measles, rubella, varicella, mumps, and influenza (flu), are preventable through vaccination. Vaccination is the most effective intervention to reduce the incidence of vaccine-preventable diseases (VPD) in infants.^3^^,^^4^ Among developed countries, Japan has been noted to have high incidence rates of VPD, such as measles, rubella, mumps, and varicella.^5^ In Japan, pertussis, measles, and rubella vaccines for infants have been included in routine vaccinations, which are principally covered by the regional government.^6^ Varicella vaccine was added to routine vaccinations in October 2014, resulting in increased vaccination rates and decreased incidence rates.^7^^,^^8^ Meanwhile, vaccines for mumps and pediatric influenza remain voluntary.^9^ Caregivers bear the costs, which has slowed the increase in vaccination rates and caused a significant disease burden.^9^^,^^10^

Systematic reviews of vaccine coverage have identified limited access to vaccines, low education levels, low literacy, and low socioeconomic status as factors contributing to vaccine non-compliance in developing countries.^11^ However, even in developed countries where vaccines are readily accessible, some parents choose not to vaccinate their children. Awareness of disease susceptibility and knowledge of vaccines and their societal impact have been identified as underlying factors.^12^ The Japan Environment and Children’s Study (JECS), a Japanese birth cohort study, has reported that being unmarried or having a lower maternal educational level was associated with under-vaccination at the age of 2 years, which was partially mediated by household income.^13^ It is speculated that parental educational background is associated with the occurrence of infectious diseases through vaccination behavior. Additionally, for vaccines not included in routine vaccination, household economic status may also be associated with non-vaccination.

Resistance to bacterial and viral infections involves activation of the host’s natural and acquired immune responses. Lifestyle habits, including nutritional intake, exercise, and sleep, affect immunity,^14^^,^^15^ which may be related to education-based health literacy. Contact with other children in daycare facilities increases the likelihood of contracting bacteria and viruses,^16^^,^^17^ and parents’ educational backgrounds may influence their decision to utilize such facilities. Exposure to chemicals, like environmental tobacco smoke, during developmental stages affects immune function.^18^^,^^19^ Parental education and socioeconomic background have been linked to household smoking in Japan and overseas.^20^^–^^22^ Thus, parental educational background may influence children’s susceptibility to infectious diseases mediated by household smoking.

In developed countries, numerous reports have linked education and socioeconomic background to the risk of infectious diseases.^23^^,^^24^ However, there are few reports on the relationship between parental educational background and childhood infectious diseases.^25^^,^^26^ Understanding factors related to children’s vaccination and home environment that influence the incidence of childhood infectious diseases contributes to public health and parental health guidance. Additionally, it provides scientific evidence for the national immunization program strategies. Therefore, this study aimed to investigate the association between parental educational background and the incidence of VPD in children up to 3 years of age using data from a nationwide birth cohort study in Japan and to elucidate how vaccination, household income, attending daycare facilities, and household smoking mediate this relationship.

## METHODS

### Participants

The participants were children from the JECS. This large nationwide birth cohort study was funded by the Ministry of Environment (MOE), Japan. The protocol and baseline data for the JECS have been described elsewhere.^27^^,^^28^ Briefly, 103,060 pregnancies were registered between January 2011 and March 2014 at 15 Regional Centers located across Japan.

**Figure 1.**
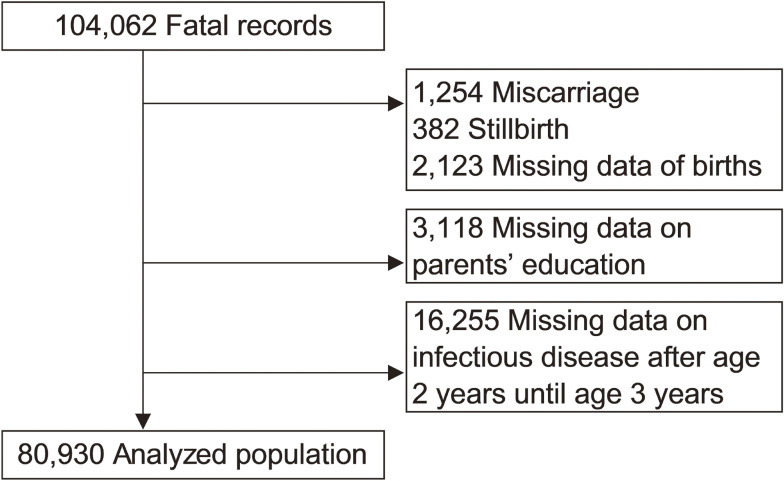
Flowchart outlining the inclusion process of Children.

This study was based on the JECS dataset jecs-ta-20190930, released in October 2019. The dataset included 104,062 fetal records. We excluded cases of miscarriage (*n* = 1,254), stillbirth (*n* = 382), missing data on births (*n* = 2,123), missing data on parents’ educational background (*n* = 3,118), and missing data on children’s infectious disease histories at 3 years of age (*n* = 16,255). The final study population included 80,930 children and their parents (Figure 1).

### Data collections

#### Outcomes

Data on children’s infectious diseases diagnosed after age 2 years were obtained from questionnaires completed by parents or caregivers at age 3 years. The infectious diseases examined included varicella, mumps, influenza, measles, rubella, and pertussis, all of which have available vaccinations in Japan.

#### Exposure

The highest levels of education of mothers and fathers were obtained via a questionnaire completed by mothers during mid or late pregnancy. Education was assessed across seven categories and reclassified into four categories based on years of education: <10 years (junior high school), 10–12 years (high school), 13–15 years (technical junior college, technical/vocational college, and associate degree), and ≥16 years (bachelor’s degree and graduate degree).

#### Covariates and intermediate variables

To minimize confounding bias, information used as covariates and intermediate variables was selected using a directed acyclic graph (eFigure 1) and obtained through self-administered questionnaires completed by parents or caregivers. In the logistic regression models, covariates included the other partner’s educational history (the four categories mentioned above) and Regional Centers (Hokkaido, Miyagi, Fukushima, Chiba, Kanagawa, Koshin, Toyama, Aichi, Kyoto, Osaka, Hyogo, Tottori, Kochi, Fukuoka, and South Kyushu/Okinawa).

In the path analyses, the following variables were used as intermediate variables between parents’ educational background and children’s infectious diseases: household smoking (rarely, sometimes, and often), annual household income (<2, ≥2 to <4, ≥4 to <6, and ≥6 million Japanese yen), attending daycare facilities at 2 years old (yes and no), and vaccinations against the corresponding diseases (yes and no). Household smoking data were obtained at 1.5 years of age by asking whether the child was exposed to cigarette smoke. Information about vaccinations for chickenpox, mumps, measles, rubella, and pertussis were obtained from ages 6 months to 2 years. Children were classified as ‘vaccinated’ if they had received at least one vaccination for the corresponding disease. Information on influenza virus vaccination was obtained only from the questionnaire at 2 years of age because the effects of influenza vaccination do not last for multiple years.

### Statistical analyses

Descriptive statistics were used to summarize the distribution of the participant characteristics. Binomial logistic regression analyses were performed using the two models to examine the association between parental education and children’s infectious diseases. The crude model was not adjusted for any covariates. The adjusted model was adjusted for the partner’s educational history and Regional Centers as covariates.

**Figure 2.**
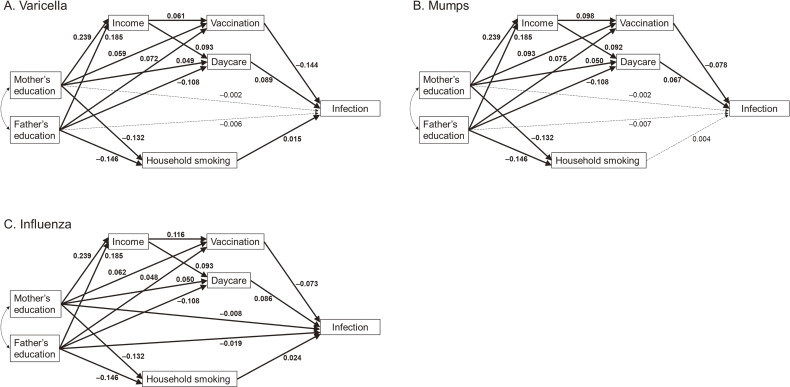
Path diagram illustrating the relationship between parents’ education level and children’s infectious diseases covered by voluntary vaccination. Solid lines indicate 95% credible intervals that do not include zero, while dashed lines indicate 95% credible intervals that include zero.

Subsequently, path analyses were performed to examine the involvement of vaccination, income, daycare attendance, and household smoking as intermediate variables in the association between parents’ education and the incidence of infectious diseases in their children. Figure 2 shows the constructed path-analysis model. Using these models, we first examined the direct effects, indirect effects (via vaccination, income, day care attendance, or household smoking), and total effects of the associations between parents’ education and children’s infection. To examine the role of income in voluntary vaccination, we compared the direct and indirect effects via income on the association between parental education and voluntary vaccination. Path analyses were performed via Bayesian estimation using the Markov chain Monte Carlo method. SPSS ver. 27 and SPSS Amos ver. 27 (IBM Corp., Armonk, NY, USA) were used for statistical analyses.

### Ethical issues

The JECS study was conducted in accordance with the Declaration of Helsinki and other internationally valid regulations and guidelines. The JECS protocol was reviewed and approved by the Ministry of the Environment’s Institutional Review Board on Epidemiological Studies (No. 100910001) and the Ethics Committees of all participating institutions. Written informed consent was obtained from all participants.

## RESULTS

Table 1 presents the characteristics of parents and children. The majority of mothers had 13–15 years of education (43.0%), while fathers had higher proportions of 10–12 years (35.7%) and ≥16 years (35.0%). Among infectious diseases covered by voluntary vaccination, the vaccination rates for varicella (until September 2014), mumps, and influenza were 64.9%, 39.5%, and 55.0%, respectively. The vaccination rates for measles, rubella, and pertussis covered by routine vaccinations were 90.2%, 87.3%, and 98.7%, respectively. The incidence rates of infections in children aged 2–3 years old were low for measles (0.0%), rubella (0.1%), and pertussis (0.1%). However, they were higher for varicella (4.5%), mumps (1.9%), and influenza (13.2%).

**Table 1.** Characteristics of parents and children (*n* = 80,930)

	*n*	(%)
*Parental characteristics*		
Mother’s years of education		
<10	3,017	(3.7)
10–12	24,332	(30.1)
13–15	34,806	(43.0)
≥16	18,775	(23.2)
Father’s years of education		
<10	5,161	(6.4)
10–12	28,869	(35.7)
13–15	18,543	(22.9)
≥16	28,357	(35.0)
Household income, million JPY/year		
<2	3,714	(4.6)
≥2 to <4	25,773	(31.8)
≥4 to <6	25,576	(31.6)
≥6	21,095	(26.1)
Missing	4,772	(5.9)
Maternal age at delivery, years, mean (SD)	31.5	(0.0)
Household smoking near children		
Almost none	60,232	(74.4)
Occasionally	15,185	(18.8)
Often	2,970	(3.7)
Missing	2,543	(3.1)
*Children’s characteristics*		
Child’s sex		
Male	41,449	(51.2)
Female	39,481	(48.8)
Multiple birth	1,441	(1.8)
Number of older sibling(s)		
None	35,063	(43.3)
≥1	45,435	(56.1)
Missing	432	(0.5)
Daycare attendance at the age of 2 years		
No	39,608	(48.9)
Yes	38,456	(47.5)
Missing	2,866	(3.5)
Vaccination until the age of 2 years		
Varicella	52,508	(64.9)
Missing	2,587	(3.2)
Mumps	31,979	(39.5)
Missing	2,902	(3.6)
Influenza	44,538	(55.0)
Missing	2,242	(2.8)
Measles	72,982	(90.2)
Missing	2,063	(2.5)
Rubella	70,660	(87.3)
Missing	2,099	(2.6)
Pertussis	79,861	(98.7)
Missing	660	(0.8)
Infections diagnosed after the age of 2 years and until the age of 3 years		
Varicella	3,625	(4.5)
Mumps	1,571	(1.9)
Influenza	10,693	(13.2)
Measles	19	(0.0)
Rubella	65	(0.1)
Pertussis	61	(0.1)

JPY, Japanese yen.

Table 2 presents the descriptive statistics of the outcomes and intermediate variables according to the parental educational background. Incidences of varicella, mumps, and influenza infections were lower among children with mothers and fathers with higher education levels. Vaccination rates against all infectious diseases were higher among children whose both mothers and fathers had higher education. Household income was higher for both mothers and fathers with higher educational levels. More educated mothers had a higher percentage of children attending daycare facilities, whereas this was lower for more educated fathers. Household smoking was more frequent among mothers and fathers with less education.

**Table 2.** Descriptive statistics of outcomes and intermediate variables by parents’ educational background

	Mother’s years of education				Father’s years of education			
	
	<10	10–12	13–15	≥16	<10	10–12	13–15	≥16
	(%)	(%)	(%)	(%)	(%)	(%)	(%)	(%)
Infections diagnosed from age 2 to 3 years								
Varicella	6.2	4.8	4.4	4.0	6.1	4.9	4.6	3.7
Mumps	2.5	2.1	2.0	1.6	2.8	2.1	2.0	1.5
Influenza	16.6	14.0	13.0	12.1	16.4	14.2	13.3	11.6
Measles	0.0	0.0	0.0	0.0	NA	0.0	0.0	0.0
Rubella	0.1	0.1	0.1	0.1	0.1	0.1	0.1	0.1
Pertussis	0.2	0.1	0.1	0.1	0.1	0.1	0.1	0.1
Vaccination status up to age 2 years								
Varicella	49.2	59.9	65.5	72.8	51.8	61.1	63.3	72.2
Mumps	19.6	31.7	41.0	50.1	24.1	34.2	38.9	48.1
Influenza	34.0	48.4	58.2	61.1	38.8	51.6	56.1	60.8
Measles	81.0	88.0	91.0	93.0	83.4	89.0	90.5	92.4
Rubella	74.8	84.0	88.4	91.6	79.3	85.4	87.8	90.4
Pertussis	95.5	98.2	99.0	99.1	97.0	98.5	98.9	99.1
Household income, million JPY/year								
<2	17.0	7.4	3.2	1.6	13.4	6.3	3.9	1.7
≥2 to <4	45.8	41.5	31.0	18.6	46.3	38.2	34.5	21.0
≥4 to <6	20.1	28.6	34.4	32.2	22.7	30.4	32.5	33.9
≥6	6.9	14.4	25.9	44.6	9.9	18.0	23.2	39.1
Daycare attendance at 2 years old								
Yes	41.0	45.1	48.8	49.3	50.9	49.2	50.2	43.4
Household smoking near children								
Almost none	50.9	66.8	75.9	85.4	51.7	68.9	75.2	83.6
Occasionally	30.5	24.1	18.3	10.8	31.9	22.8	18.8	12.2
Often	12.1	5.5	2.9	1.3	11.5	4.8	3.0	1.5

JPY, Japanese yen; NA, not applicable.

Table 3 shows the odds ratios of child infection using logistic regression models, with education of 10–12 years as the reference. For varicella, mumps, and influenza, both mothers and fathers with less than 10 years of education had higher odds ratios, whereas odds ratios were lower for education levels of ≥16 years. For influenza, fathers with 13–15 years of education also had lower odds ratios (0.94; 95% confidence interval [CI], 0.89–0.99 in the adjusted model). For measles, rubella, and pertussis, the odds ratio for pertussis was higher for mothers with less than 10 years of education (2.62; 95% CI, 1.01–6.84). Lower odds ratios for measles, rubella, and pertussis were observed in both parents with longer education, albeit not statistically significant.

**Table 3.** Association of parental education with child’s infection from age 2 to 3 years estimated using logistic regression models

			Mother’s years of education				Father’s years of education			
	
			<10	10–12	13–15	≥16	<10	10–12	13–15	≥16
Varicella	Crude model	cOR	**1.31**	1.00	0.91	**0.83**	**1.25**	1.00	0.93	**0.74**
		(95% CI)	**(1.12**–**1.54)**	[Reference]	(0.84–0.98)	**(0.75–0.91)**	**(1.11–1.42)**	[Reference]	(0.86–1.02)	**(0.68–0.80)**
	Adjusted model^a^	aOR	**1.22**	1.00	0.96	0.98	**1.20**	1.00	0.96	**0.78**
		(95% CI)	**(1.04–1.43)**	[Reference]	(0.89–1.04)	(0.88–1.08)	**(1.05–1.36)**	[Reference]	(0.88–1.05)	**(0.72–0.86)**

Mumps	Crude model	cOR	1.18	1.00	0.94	**0.73**	**1.34**	1.00	0.96	**0.71**
		(95% CI)	(0.92–1.51)	[Reference]	(0.83–1.05)	**(0.64–0.85)**	**(1.11–1.61)**	[Reference]	(0.84–1.09)	**(0.63–0.80)**
	Adjusted model^a^	aOR	1.02	1.00	0.95	**0.83**	**1.25**	1.00	0.93	**0.73**
		(95% CI)	(0.79–1.31)	[Reference]	(0.84–1.07)	**(0.71–0.97)**	**(1.04–1.51)**	[Reference]	(0.81–1.06)	**(0.64–0.83)**

Influenza	Crude model	cOR	**1.22**	1.00	**0.92**	**0.85**	**1.18**	1.00	**0.93**	**0.79**
		(95% CI)	**(1.10–1.36)**	[Reference]	**(0.88–0.97)**	**(0.80–0.90)**	**(1.09–1.28)**	[Reference]	**(0.88–0.98)**	**(0.75–0.83)**
	Adjusted model^a^	aOR	**1.14**	1.00	0.96	0.94	**1.13**	1.00	**0.94**	**0.80**
		(95% CI)	**(1.03–1.27)**	[Reference]	(0.91–1.01)	(0.89–1.00)	**(1.04–1.23)**	[Reference]	**(0.89–0.99)**	**(0.76–0.85)**

Measles	Crude model	cOR	0.90	1.00	0.39	0.58	NA	1.00	0.71	0.28
		(95% CI)	(0.11–7.08)	[Reference]	(0.13–1.16)	(0.18–1.87)		[Reference]	(0.25–2.04)	(0.08–1.00)
	Adjusted model^a^	aOR	1.05	1.00	0.42	0.85	NA	1.00	0.82	0.31
		(95% CI)	(0.13–8.31)	[Reference]	(0.14–1.28)	(0.24–3.06)		[Reference]	(0.27–2.42)	(0.08–1.22)

Rubella	Crude model	cOR	0.97	1.00	0.75	0.52	1.35	1.00	0.64	0.60
		(95% CI)	(0.29–3.21)	[Reference]	(0.44–1.30)	(0.25–1.08)	(0.59–3.08)	[Reference]	(0.33–1.26)	(0.33–1.09)
	Adjusted model^a^	aOR	0.89	1.00	0.90	0.71	1.36	1.00	0.71	0.77
		(95% CI)	(0.26–3.02)	[Reference]	(0.51–1.59)	(0.32–1.58)	(0.59–3.17)	[Reference]	(0.36–1.42)	(0.40–1.49)

Pertussis	Crude model	cOR	**2.85**	1.00	1.15	0.76	1.40	1.00	0.84	0.76
		(95% CI)	**(1.12–7.23)**	[Reference]	(0.63–2.10)	(0.35–1.66)	(0.57–3.42)	[Reference]	(0.43–1.66)	(0.41–1.41)
	Adjusted model^a^	aOR	**2.62**	1.00	1.20	0.85	1.14	1.00	0.86	0.91
		(95% CI)	**(1.01–6.84)**	[Reference]	(0.65–2.24)	(0.36–1.98)	(0.51–3.41)	[Reference]	(0.43–1.72)	(0.47–1.76)

aOR, adjusted odds ratio; CI, confidence interval; cOR, crude odds ratio; NA, not applicable.

^a^Adjusted Model included mother’s education, father’s education, and regional centers.

Bold letters indicate 95% CIs that do not include 1.

**Figure 3.**
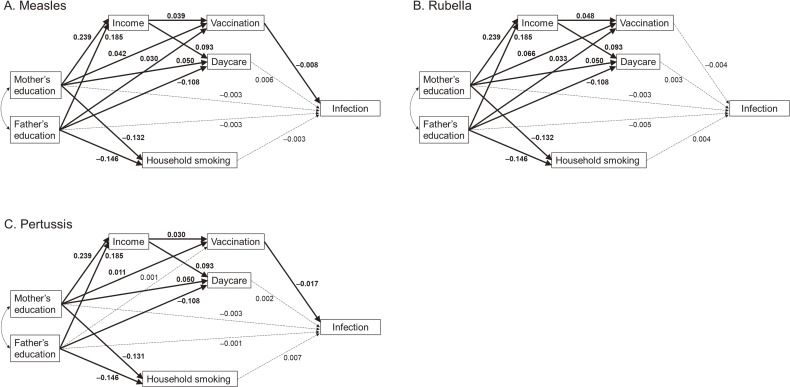
Path diagram illustrating the relationship between parents’ education level and children’s infectious diseases covered by routine vaccination. Solid lines indicate 95% credible intervals that do not include zero, while dashed lines indicate 95% credible intervals that include zero.

Figure 2, Figure 3, and eTable 1 show the standardized β values for the associations between parental education and infection, as well as the mediating variables of vaccination, household income, daycare attendance, and household smoking. Maternal education level showed a positive association with daycare attendance, whereas paternal education level showed a negative association.

Vaccinations for varicella, mumps, and influenza were negatively associated with each respective infection. However, daycare attendance was positively associated with infections. Household smoking was positively associated with varicella and influenza infections. Regarding influenza, the level of parental education was negatively associated with infection. Vaccinations for measles and pertussis were negatively associated with each respective infection; however, the effect sizes were small.

Table 4 shows the total, direct, and indirect effects (sum of all indirect effects through income, vaccination, daycare, and household smoking) for the association of parental education with children’s infections using path analyses. For varicella, parental education had negative indirect effects for child infection (β −0.006; 95% Bayesian credible interval [BCI], −0.008 to −0.004 for mothers and −0.022; 95% BCI, −0.024 to −0.020 for fathers) and a negative total effect (−0.008; 95% BCI, −0.015 to −0.002 for mothers and −0.029; 95% BCI, −0.036 to −0.021 for fathers). For mumps, parental education had negative indirect effects (−0.005; 95% BCI, −0.006 to −0.003 for mothers and −0.014; 95% BCI, −0.016 to −0.012 for fathers), and fathers’ education had a negative total effect (−0.021; 95% BCI, −0.029 to −0.014). For influenza, parental education had direct effects (−0.008; 95% BCI, −0.016 to −0.001 for mothers and −0.019; 95% BCI, −0.026 to −0.011 for fathers), indirect effects (−0.004; 95% BCI, −0.005 to −0.002 for mothers and −0.016; 95% BCI, −0.018 to −0.015 for fathers), and total effect (−0.012; 95% BCI, −0.020 to −0.005 for mothers and −0.035; 95% BCI, −0.043 to −0.028 for fathers). Fathers’ education was more strongly associated with these infections than mothers’ education. No associations were found between parental education and child infections with measles, rubella, or pertussis.

**Table 4.** Association of parental education with child’s infection from age 2 to 3 years estimated by the path analyses

Child infection	Standardized β (95% BCI)											

	Mother’s education						Father’s education					

	Total effect		Direct effect		Indirect effect^a^		Total effect		Direct effect		Indirect effect^a^	
Varicella	−**0.008**	**(−0.015 to −0.002)**	−0.002	(−0.009 to 0.006)	**−0.006**	**(−0.008 to −0.004)**	**−0.029**	**(−0.036 to −0.021)**	−0.006	(−0.014 to 0.001)	**−0.022**	**(−0.024 to −0.020)**
Mumps	−0.007	(−0.015 to 0.000)	−0.002	(−0.010 to 0.005)	**−0.005**	**(−0.006 to −0.003)**	**−0.021**	**(−0.029 to −0.014)**	−0.007	(−0.015 to 0.000)	**−0.014**	**(−0.016 to −0.012)**
Influenza	**−0.012**	**(−0.020 to −0.005)**	**−0.008**	**(−0.016 to −0.001)**	**−0.004**	**(−0.005 to −0.002)**	**−0.035**	**(−0.043 to −0.028)**	**−0.019**	**(−0.026 to −0.011)**	**−0.016**	**(−0.018 to −0.015)**
Measles	−0.003	(−0.011 to 0.005)	−0.003	(−0.011 to 0.004)	0.000	(−0.001 to 0.002)	−0.003	(−0.011 to 0.004)	−0.003	(−0.011 to 0.005)	−0.001	(−0.002 to 0.001)
Rubella	−0.004	(−0.011 to 0.004)	−0.003	(−0.011 to 0.004)	−0.001	(−0.002 to 0.001)	−0.006	(−0.014 to 0.001)	−0.005	(−0.013 to 0.002)	−0.001	(−0.002 to 0.000)
Pertussis	−0.004	(−0.012 to 0.003)	−0.003	(−0.011 to 0.004)	−0.001	(−0.002 to 0.000)	−0.003	(−0.010 to 0.005)	−0.001	(−0.009 to 0.007)	−0.001	(−0.003 to 0.000)

BCI, Bayesian credible interval.

^a^Via income, vaccination, daycare attendance, and household smoking.

Bold letters indicate 95% BCIs that do not include zero.

Table 5 compares the direct and indirect effects of household income on the association of parental education with varicella, mumps, and influenza vaccinations. For all vaccinations, both direct and indirect effects were observed; however, the association between parental education and vaccination was stronger for the direct effects than for the indirect effects via income.

**Table 5.** Comparison of the association of parental education with child’s voluntary vaccination between direct effects (non-income-mediated path) and indirect effects (income-mediated path) by the path analyses

Vaccination	Standardized β (95% BCI)							

	Mother’s education				Father’s education			
	
	Direct effect (non-income-mediated)		Indirect effect (income-mediated)		Direct effect (non-income-mediated)		Indirect effect (income-mediated)	
Varicella	**0.059**	**(0.051–0.066)**	**0.015**	**(0.013–0.017)**	**0.072**	**(0.064–0.080)**	**0.011**	**(0.010–0.013)**
Mumps	**0.093**	**(0.085–0.101)**	**0.023**	**(0.022–0.026)**	**0.075**	**(0.067–0.083)**	**0.018**	**(0.017–0.020)**
Influenza	**0.062**	**(0.055–0.070)**	**0.028**	**(0.026–0.030)**	**0.048**	**(0.040–0.055)**	**0.021**	**(0.020–0.023)**

BCI, Bayesian credible interval.

Bold letters indicate 95% BCIs that do not include zero.

## DISCUSSION

This study investigated the relationship between parental education and the incidence of VPD, specifically varicella, mumps, influenza, measles, rubella, and pertussis, which are common in children. The study revealed that: 1) there were negative associations between parental education level and the incidence of VPD, such as varicella, mumps, and influenza, where vaccination rates were low; 2) these associations were mediated by vaccination, household income, daycare attendance, and household smoking; and 3) there were both income-mediated and non-income-mediated pathways between parental education and voluntary vaccination.

Japan’s immunization schedule consists of routine and voluntary immunizations.^6^ The difference between the two lies in the family’s financial burden for the vaccinations. Routine vaccines are mainly paid for by local governments, and parents do not need to bear the cost. The differences in child immunization and infectious disease incidence rates in this study reflect the differences between the two immunization strategies. Of the six typical infectious diseases among the children examined in this study, measles, rubella, and pertussis were covered by routine vaccinations. Vaccination rates for these infections in this study were high (87.3–98.7%), and infection rates were very low (0.0–0.1%). In Japan, measles and rubella vaccines are given simultaneously as a combined vaccine called the “MR vaccine.” Therefore, it was assumed that the vaccination rates for both vaccines would be similar. However, according to the parents’ responses, the rubella vaccination rate was lower than that of measles. This difference was more pronounced among mothers with lower levels of education, suggesting a possible lack of understanding regarding the content of the combined vaccine, particularly the inclusion of the rubella vaccine.

Voluntary vaccination programs in Japan cover vaccination against mumps and influenza. The varicella vaccine was added to the routine immunization schedule for children 12 to 36 months of age in October 2014. This study included children born between 2011 and 2014; thus, some were eligible for routine varicella vaccination. The study reported a varicella vaccination rate of 64.9% and an infection incidence rate of 4.5%. For mumps and influenza, the vaccination rates were 39.5% and 55.0%, respectively, and the infection rates were 1.9% and 13.2%, respectively. The World Health Organization recommends that immunization programs be considered for mumps, varicella, and seasonal influenza.^29^^–^^31^ The results of this study on vaccination and infection rates may provide a basis for the need for routine vaccination in the prevention of infectious diseases in children. In diseases with low vaccination rates, such as varicella, mumps, and influenza, higher levels of parental education were associated with a lower incidence of child infections. Path analysis revealed that this association was mediated by vaccination, income, daycare attendance, and household smoking. In the path through vaccination, higher parental education levels were associated with higher vaccination rates, which, in turn, were associated with lower infection rates. Furthermore, both direct effects (non-income-mediated) and indirect effects (income-mediated) were observed in the association between parents’ education levels and children’s voluntary vaccination. The indirect effects via income suggest that the cost burden of vaccination contributes to lower vaccination coverage. According to a survey conducted in Kanazawa (2019–2020), the most common reason parents cited for not vaccinating their children against mumps was that it was not a routine vaccination (35.9%), while only 2.2% expressed concern about adverse reactions.^32^ Vaccination coverage for the varicella vaccine has increased significantly since it became a routine vaccination in 2014, reaching 90% in 2017, up from approximately 20% in the 1990s.^8^ Therefore, including these vaccines in routine vaccination programs could increase vaccination coverage and reduce income disparities. However, as shown in Table 5, the direct effects (non-income-mediated) between parental education level and voluntary vaccination were stronger than the indirect effects (income-mediated) for all three diseases. This finding suggests that factors other than economic circumstances substantially influence non-vaccination of children. This may be primarily related to parental attitudes, known as vaccination hesitancy, which can stem from concerns about vaccine safety and efficacy, inadequate awareness of vaccine benefits, and distrust towards governments and health professionals, and should be addressed via a multifaceted educational approach.^33^ A systematic review reported that parental perceptions of children’s susceptibility to disease were associated with their children’s vaccination, and perceptions of less severe illness or less frequent complications were associated with non-vaccination.^12^ Japanese studies have reported that having a general understanding of vaccination, reading information from government sources, and receiving recommendations from family and doctors are positive factors for mumps vaccination, whereas, the perceptions of vaccines ineffectiveness and fear of side effects are negative factors.^32^^,^^34^ Therefore, to reduce the incidence of these infectious diseases in children, it would be worthwhile to disseminate correct and transparent information about infectious diseases and vaccination that is easily understood by parents and families across all educational levels. For example, vaccination recommendations can be provided when distributing the maternal and child health handbooks to all expectant mothers during prenatal daycare classes, post-delivery examinations by primary care physicians and midwives, regular checkups, and routine immunizations for children. It will be crucial for health professionals to establish trust with parents and provide information that addresses parents’ concerns about vaccines. Additionally, the internet has become a major source for health information, although identifying reliable sources can be challenging. Therefore, disseminating information that addresses parents’ vaccination hesitancy through credible websites may be beneficial.

The path through household smoking indicates that parents with higher education levels smoked less frequently near their children, which was weakly associated with lower incidence of varicella and influenza infections. This is consistent with reports linking current smoking to higher rates of influenza infection in adults, likely due to increased susceptibility to infection and decreased immune response, as well as structural changes in the respiratory tract.^35^^,^^36^ Passive smoking in children has also been reported to be associated with influenza infection and influenza-related hospitalizations.^37^^,^^38^ Our study showed that passive smoking may also affect the susceptibility of children to varicella infection.

Children’s attendance at daycare was positively associated with varicella, mumps, and influenza infections. However, contrasting patterns were observed in parental education levels regarding daycare attendance: higher maternal education levels were correlated with increased daycare usage by the child, whereas higher paternal education levels were associated with decreased daycare usage. It is hypothesized that as maternal education increases, so does employment, leading to higher daycare attendance rates. Conversely, higher paternal education often correlates with lower maternal employment rates, prompting choices for home-based daycare. Consequently, higher paternal education was associated with fewer infections, influenced by increased vaccination, reduced household smoking, and less daycare attendance. In contrast, higher maternal education was linked to increased infections due to greater daycare use, offsetting the effects of vaccination and reduced household smoking, resulting in minimal or no overall infection association.

In addition to the indirect effects through the aforementioned intermediate variables, weak direct effects were observed for parents’ education levels and children’s influenza infection. This effect may have been mediated by factors that were not addressed in this study. For example, hygiene practices, such as hand washing, may also be related to parental education levels.

Regarding measles, rubella, and pertussis covered by routine vaccination, no association with education level was observed for either mothers or fathers. There was only a weak negative association between vaccination and the incidence of measles and pertussis. This is attributed to high vaccination rates that achieve herd immunity among young children, effectively preventing infection.^39^^,^^40^ However, even for routine vaccination, associations have been observed between higher education levels and higher vaccination rates.

The strength of this study lies in the use of a large sample size from the birth cohort study JECS, which allowed for a highly accurate analysis. Additionally, this study examined the association of parental education with six infectious diseases covered by voluntary or routine vaccinations, enabling a comparison between the two. However, this study had several limitations. First, we could not adjust for other potential mediating or confounding factors that were not investigated, such as parental attitudes toward vaccination. Second, the incidence of infectious diseases and administered vaccination were based on parental responses to questionnaires, which may have been subject to recall bias. Third, it is not clear whether children received the number of vaccinations required by the immunization program. Fourth, this study was conducted using the birth cohort study in Japan, and the results may not be generalizable to regions with different educational systems or vaccination programs. Fifth, while this study revealed an association between parental education and the six major VPD in children, the association may differ for other infectious diseases.

### Conclusion

Parents’ educational levels were associated with lower infection rates of varicella, mumps, and influenza, which are covered by voluntary vaccination in Japan. Both income-mediated and non-income-mediated pathways may influence the association between parental educational and vaccination rates. Enhancing communication about vaccination benefits across all educational levels, alongside financial support, could reduce childhood infectious diseases. Future studies that adjust for other potential mediating factors and account for recall bias may help validate our findings.

## SUPPLEMENTARY MATERIAL

eTable 1. Direct effects between all variables in the path analysis

eFigure 1. Directed acyclic graph for the association between parental education and child infection
