# Supplementary material for: Associations of Parental Education With Children’s Infectious Diseases and Their Mediating Factors: The Japan Environment and Children’s Study (JECS)
Source: J Epidemiol. 2025 Apr 5;35(4):178–86. doi: 10.2188/jea.JE20240192 (PMC11882350; doi:10.2188/jea.JE20240192)
Supplement: Supplementary file 1 [file je-35-178-s001.pdf]

eTable 1. Direct effects between all variables in the path analysis

		Standardized β (95% BCI)											
		Mother's education		Father's education		Income		Daycare		Household smoking		Vaccination	
Varicella	Income	**0.239**	**(0.232–0.246)**	**0.185**	**(0.177–0.192)**	–		–		–		–	
	Daycare	**0.049**	**(0.042–0.057)**	**-0.108**	**(-0.115 to -0.100)**	**0.093**	**(0.085–0.100)**	–		–		–	
	Household smoking	**-0.132**	**(-0.139 to -0.125)**	**-0.146**	**(-0.154 to -0.139)**	–		–		–		–	
	Vaccination	**0.059**	**(0.051 to 0.066)**	**0.072**	**(0.064 to 0.080)**	**0.061**	**(0.053–0.069)**	–		–		–	
	Infection	-0.002	(-0.009 to 0.006)	-0.006	(-0.014 to 0.001)	–		**0.089**	**(0.082–0.096)**	**0.015**	**(0.008–0.022)**	**-0.144**	**(-0.151 to -0.136)**
Mumps	Income	**0.239**	**(0.232–0.246)**	**0.185**	**(0.177–0.192)**	–		–		–		–	
	Daycare	**0.050**	**(0.041–0.058)**	**-0.108**	**(-0.116 to -0.100)**	**0.092**	**(0.085–0.100)**	–		–		–	
	Household smoking	**-0.132**	**(-0.139 to -0.124)**	**-0.146**	**(-0.154 to -0.139)**	–		–		–		–	
	Vaccination	**0.093**	**(0.085–0.101)**	**0.075**	**(0.067–0.083)**	**0.098**	**(0.091–0.106)**	–		–		–	

	Infection	-0.002	(-0.010 to 0.005)	-0.007	(-0.015 to 0.000)	–		**0.067**	**(0.060–0.074)**	0.004	(-0.002 to 0.011)	**-0.078**	**(-0.085 to -0.071)**
Influenza	Income	**0.239**	**(0.232–0.246)**	**0.185**	**(0.178–0.192)**	–		–		–		–	
	Daycare	**0.050**	**(0.042–0.057)**	**-0.108**	**(-0.116 to -0.100)**	**0.093**	**(0.085–0.100)**	–		–		–	
	Household smoking	**-0.132**	**(-0.139 to -0.124)**	**-0.146**	**(-0.154 to -0.139)**	–		–		–		–	
	Vaccination	**0.062**	**(0.055–0.070)**	**0.048**	**(0.040–0.055)**	**0.116**	**(0.109–0.124)**	–		–		–	
	Infection	**-0.008**	**(-0.016 to 0.001)**	**-0.019**	**(-0.026 to -0.011)**	–		**0.086**	**(0.079–0.093)**	**0.024**	**(0.017–0.031)**	**-0.073**	**(-0.080 to -0.066)**
Measles	Income	**0.239**	**(0.232–0.247)**	**0.185**	**(0.177–0.192)**	–		–		–		–	
	Daycare	**0.050**	**(0.042–0.057)**	**-0.108**	**(-0.115 to -0.100)**	**0.093**	**(0.085–0.100)**	–		–		–	
	Household smoking	**-0.132**	**(-0.139 to -0.124)**	**-0.146**	**(-0.154 to -0.139)**	–		–		–		–	
	Vaccination	**0.042**	**(0.034–0.050)**	**0.030**	**(0.022–0.038)**	**0.039**	**(0.031–0.047)**	–		–		–	
	Infection	-0.003	(-0.011 to 0.004)	-0.003	(-0.011 to 0.005)	–		0.006	(-0.001 to 0.013)	-0.003	(-0.010 to 0.005)	**-0.008**	**(-0.015 to -0.001)**

Rubella	Income	**0.239**	**(0.232–0.247)**	**0.185**	**(0.177–0.192)**	–		–		–		–	
	Daycare	**0.050**	**(0.042–0.057)**	**-0.108**	**(-0.115 to -0.100)**	**0.093**	**(0.085–0.100)**	–		–		–	
	Household smoking	**-0.132**	**(-0.139 to -0.124)**	**-0.146**	**(-0.154 to -0.139)**	–		–		–		–	
	Vaccination	**0.066**	**(0.059–0.074)**	**0.033**	**(0.025–0.040)**	**0.048**	**(0.041–0.056)**	–		–		–	
	Infection	-0.003	(-0.011 to 0.004)	-0.005	(-0.013 to 0.002)	–		0.003	(-0.004 to 0.011)	0.004	(-0.003 to 0.011)	-0.004	(-0.011 to 0.003)
Pertussis	Income	**0.239**	**(0.232–0.246)**	**0.185**	**(0.177–0.192)**	–		–		–		–	
	Daycare	**0.050**	**(0.042–0.058)**	**-0.108**	**(-0.116 to -0.100)**	**0.093**	**(0.085–0.100)**	–		–		–	
	Household smoking	**-0.131**	**(-0.139 to -0.124)**	**-0.146**	**(-0.154 to -0.139)**	–		–		–		–	
	Vaccination	**0.011**	**(0.003–0.019)**	0.001	(-0.007 to 0.009)	**0.030**	**(0.023–0.038)**	–		–		–	
	Infection	-0.003	(-0.011 to 0.004)	-0.001	(-0.009 to 0.007)	–		0.002	(-0.005 to 0.009)	0.007	(0.001 to 0.014)	**-0.017**	**(-0.024 to -0.010)**

BCI, Bayesian credible interval.

– indicates no direct path. Bold letters indicate 95% BCIs that do not include zero.

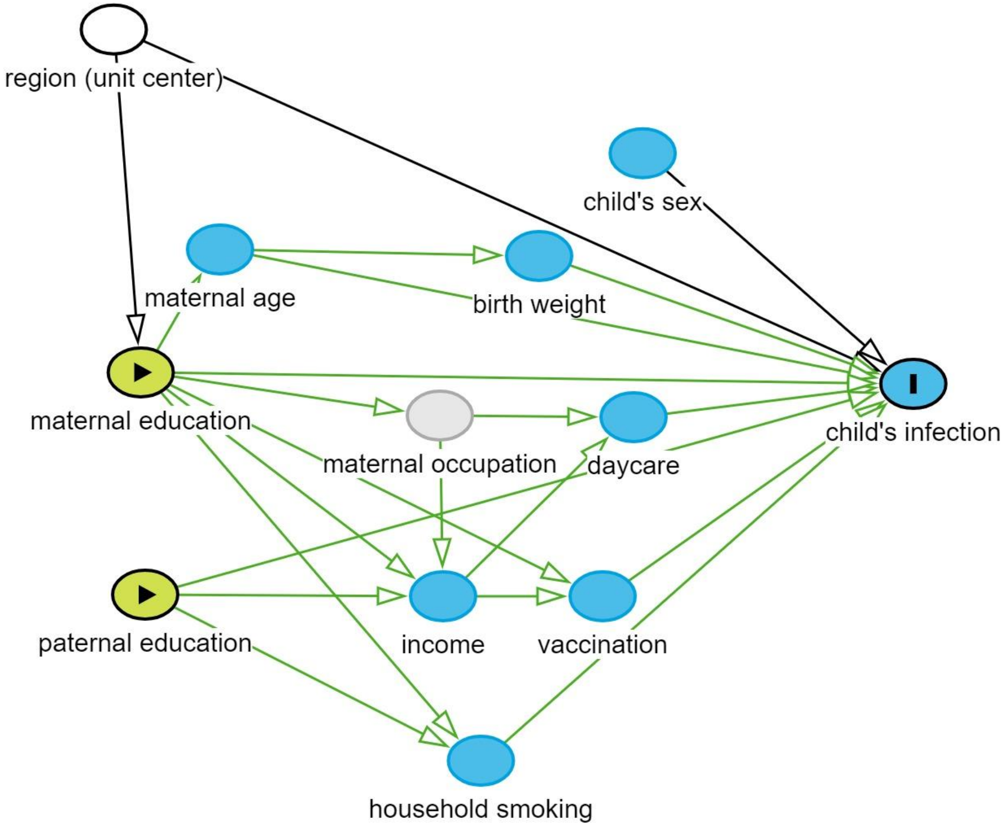

eFigure 1. Directed acyclic graph for the association between parental education and child infection
